# Pushing the Limits of Medical Management in HCM: A Review of Current Pharmacological Therapy Options

**DOI:** 10.3390/ijms22137218

**Published:** 2021-07-05

**Authors:** Cristian Stătescu, Ștefana Enachi, Carina Ureche, Laura Țăpoi, Larisa Anghel, Delia Șalaru, Carmen Pleșoianu, Mădălina Bostan, Dragoș Marcu, Mircea Ovanez Balasanian, Radu Andy Sascău

**Affiliations:** 1 Cardiology Department, Cardiovascular Diseases Institute “Prof. Dr. George I.M. Georgescu”, Carol I Boulevard No. 50, 700503 Iași, Romania; cstatescu@gmail.com (C.S.); carina.ureche@yahoo.com (C.U.); laura.tapoi@yahoo.com (L.Ț.); larisa.anghel@umfiasi.ro (L.A.); deliasalaru@gmail.com (D.Ș.); carmenplesoianu@gmail.com (C.P.); madalina_farima@yahoo.com (M.B.); dragos.marcu11@yahoo.com (D.M.); ovanes718@yahoo.com (M.O.B.); radu.sascau@gmail.com (R.A.S.); 2 Internal Medicine Department, “Grigore T. Popa” University of Medicine and Pharmacy, 700115 Iasi, Romania

**Keywords:** hypertrophic cardiomyopathy, pharmacotherapy, drug trials, myosin inhibitors, mavacamten

## Abstract

Hypertrophic cardiomyopathy (HCM) is the most common monogenic cardiac disease with a highly variable phenotypic expression, ranging from asymptomatic to drug refractory heart failure (HF) presentation. Pharmacological therapy is the first line of treatment, but options are currently limited to nonspecific medication like betablockers or calcium channel inhibitors, with frequent suboptimal results. While being the gold standard practice for the management of drug refractory HCM patients, septal reduction therapy (SRT) remains an invasive procedure with associated surgical risks and it requires the expertise of the operating centre, thus limiting its accessibility. It is therefore with high interest that researchers look for pharmacological alternatives that could provide higher rates of success. With new data gathering these past years as well as the development of a new drug class showing promising results, this review provides an up-to-date focused synthesis of existing medical treatment options and future directions for HCM pharmacological treatment.

## 1. Introduction

Hypertrophic cardiomyopathy (HCM) is the most common monogenic disorder of cardiac myocytes, characterized by left ventricular hypertrophy unexplained by secondary causes, with a nondilated left ventricle (LV) and a preserved or increased ejection fraction. Dynamic left ventricular outflow tract (LVOT) obstruction is present in approximatively two thirds of cases (at rest or provoked), being the main determinant of symptoms and causing patients to experience debilitating limitations in every day physical activity. It is most frequently secondary to a mitral-valve systolic anterior motion with or without septal contact that is produced by flow drag. Other mechanisms such as anomalous insertion of the papillary muscles can be responsible for obstruction at a midventricular level. [1,2] Mutations in over a dozen genes encoding proteins of thick and thin myofilament contractile components of the cardiac sarcomere or Z disk have been proven to cause HCM, with MYH7, encoding myosin heavy chain β (MHC-β), and MYBPC3, encoding cardiac myosin-binding protein C (cMyBP-C), being responsible for 50–70% of inherited HCM. These genetic variants are, however, found in only about one third of patients with HCM [3,4,5]. Moreover, recent data suggests that patients with pathogenic or likely pathogenic variants exhibit clinical manifestations at an earlier age with a greater risk of developing adverse outcomes compared with patients with nonfamilial HCM [6]. At a histological and morphological level this results in myocardial disarray and hypertrophy with interstitial fibrosis that further expresses itself into a state of hyperdynamic contraction and impaired relaxation.

The phenotypic expression is highly variable, ranging from asymptomatic to drug refractory heart failure (HF) presentation. Sudden cardiac death, even though infrequent (at approximatively 1% per year), remains the most dreaded complication in HCM, being caused by ventricular arrythmias due to overactivity secondary to LVOT obstruction, myocardial ischemia and cardiomyocytes disarray [5]. Constant attempts are being made in order to obtain a more accurate risk stratification, as patients considered at high risk present an indication of implantation of a defibrillator as a primary prevention measure [7,8]. Studies have shown that the incidence of ventricular arrythmias declines with age, whereas the risk for HF and atrial fibrillation increases, with data suggesting that mortality in HCM patients is predominantly determined by HF and noncardiac death and less commonly by lethal arrythmias, thus highlighting the need for lifelong surveillance and for age and risk stratified management [6].

According to American College of Cardiology Foundation/American Heart Association and European Society of Cardiology guidelines septal reduction therapy (SRT) remains the gold standard practice for the management of patients with HCM refractory to medical treatment. In spite of the benefits it can provide, it remains an invasive procedure with associated surgical risks, and it requires the expertise of the operating centre, which limits its accessibility [7,8]. Additionally, there is a small subgroup of patients that fail to experience an improvement despite surgical relief of outflow obstruction, as shown by a study on 503 patients that underwent myomectomy for drug refractory HCM, in which massive hypertrophy (≥30 mm; *p* < 0.01) and younger age (40 ± 13 years in nonresponders vs. 53 ± 14 years in responders; *p* < 0.001) were found to be the most significant predictors of nonresponsiveness [9]. Alcohol septal ablation is an alternative in patients in whom surgery is contraindicated due to comorbidities or advanced age, but it is a procedure that requires appropriate coronary anatomy, it is associated with greater risk of conduction block and with a greater need for repeat intervention due to residual gradient [8].

In the context of new data gathering these past years regarding pharmacological management of HCM as well as the development of a new drug class showing promising results, this review provides an up-to-date focused synthesis of existing medical treatment options and future directions.

## 2. Current Practice and Recent Attempts

Pharmacological therapy is the first line of treatment for patients with HCM. There is currently no targeted therapy, therefore options are limited to non-specific classes such as nonvasodilating betablockers, non-dihydropyridine calcium channel blockers and disopyramide as a second line option. These drugs can offer a variable level of symptomatology reduction, at the cost of possible adverse effects. Moreover, they do not halt disease progression [10].

**Figure 1:**
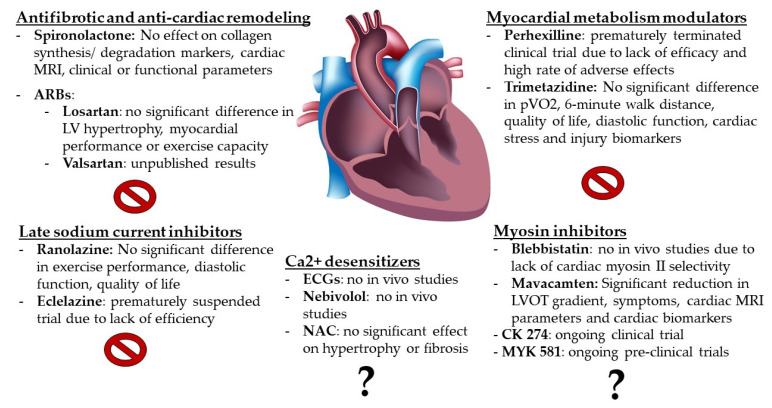
Targeted mechanisms of pharmacological therapy for HCM. ARBs = angiotensin II receptor blockers; ECGs = Epigallocatechin-3-gallate; LV = left ventricle; LVOT = left ventricle outflow tract; MRI = magnetic resonance imaging; NAC = N-acetylcysteine; pVO2 = peak oxygen consumption.

In the last decade, there have been multiple attempts to evaluate if various drugs could be repurposed if proved beneficial in the treatment of HCM (Figure 1).

By modulating myocardial fibrosis, it has been speculated that Spironolactone could improve left ventricular remodelling. However, a randomised trial conducted by Maron et al. failed to prove any benefit on serum markers of collagen synthesis or degradation, cardiac MRI, or clinical and functional parameters [11].

Another molecule known to mediate myocardial hypertrophy and fibrosis is angiotensin II, which is why studies have been performed in order to investigate if angiotensin II receptor blockers (ARBs) could halt morphological disease progression [12,13]. Initial results from a randomised pilot study that compared administration of Losartan (an ARB) versus placebo in patients with non-obstructive HCM were encouraging, showing a significant difference in the percent change in LV mass (mean change +5% [−4% to +21%] with placebo vs. −5% [−11% to −0.9%] with Losartan; *p* = 0.06) and in the extent of late gadolinium enhancement at MRI (+31% ± 26% with placebo vs. −23% ± 45% with Losartan; *p* = 0.03) [14]. Despite this fact, larger trials failed to prove significant differences in LV hypertrophy (mean difference 1 g/m^2^, 95% CI −3 to 6; *p* = 0.60) irrespective of obstructive physiology [15], as well as in myocardial performance (mean difference for left ventricular ejection fraction (LVEF) 0% (95% CI −3% to 4%), *p* = 0.84, global longitudinal strain 0.7% (95% CI −0.2% to 1.6%), *p* = 0.13) or exercise capacity (mean difference −0.3 metabolic equivalents (95% CI −1.0 to 0.3 METS), *p* = 0.28) [16]. However, the largest to date randomized, double-blinded, placebo-controlled study that tests if Valsartan could attenuate disease progression in HCM if administered early is yet to publish its results (VANISH NCT01912534) [17].

Ranolazine, a late sodium current inhibitor that reduces intracellular calcium overload, had shown positive effects on diastolic function and arrhythmic propensity in HCM cardiomyocytes in vitro and in vivo [18,19]. Nevertheless, benefits could not be translated into clinical human trials, as shown by Olivetto et al. in a phase II study that evaluated the Efficacy of Ranolazine in Patients with Symptomatic Hypertrophic Cardiomyopathy (the RESTYLE-HCM study). No difference between ranolazine and placebo was noted on exercise performance (median change in peak VO_2_ of 0.15 ± 3.96 with ranolazine versus -0.02±4.25 mL/kg per minute with placebo; *p* = 0.832), plasma brain natriuretic peptide levels (geometric mean median (interquartile range), −3 pg/mL (−107, 142 pg/mL) in ranolazine group versus 78 pg/mL (−71, 242 pg/mL) with placebo; *p* = 0.251), diastolic function, or quality of life of symptomatic patients with nonobstructive HCM [20]. Another large randomized trial (LIBERTY-HCM, NCT02291237) designed to assess a molecule of the same class, Eleclazine, was prematurely ended as preliminary data found it to be ineffective in patients with symptomatic HCM [21].

Studies have previously suggested that an inefficient deployment of adenosine triphosphate (ATP) is present in the myocardium of patients with HCM, as a reduction of the resting ratio of cardiac phosphocreatin to ATP has been observed in patients with sarcomeric mutations. This leads to an increase in myocardial demands for force production, which furtherly results in cardiac hypertrophy as a compensatory mechanism [22].

This observation led to the idea of evaluating if drugs that target myocardial metabolism such as Perhexiline (an oral inhibitor of carnitine palmitoyltransferase I) and Trimetazidine (a reversible competitive inhibitor of 3-ketoacyl-coenzyme A thiolase) could be a valuable option for patients with HCM. Despite favorable results from a pre-clinical study [23] and a phase II trial (METAL-HCM) [24] showing improvement in exercise capacity in patients treated with Perhexilline, a larger phase multicentric trial (NCT02862600) was prematurely terminated due to lack of efficacy and a high rate of adverse effects.

A randomized, placebo-controlled study meant to determine the effect of oral therapy with trimetazidine on exercise capacity in patients with symptomatic nonobstructive HCM similarly showed no significant differences regarding peak oxygen consumption, the 6-minute walk distance, quality of life, frequency of ventricular ectopic beats, diastolic function, serum N-terminal pro-brain natriuretic peptide (NT-proBNP) level or troponin T level [25].

Due to the fact that an increase in myofilament calcium (Ca^2+)^ sensitivity has been shown to be associated to HCM, thus contributing to the impaired relaxation and diastolic dysfunction, Ca^2+^ desensitizing agents have submerged as an attractive therapeutic option. Even more so if we consider that they may also have the potential ability to prevent arrhythmias in HCM patients [26,27].

Epigallocatechin-3-gallate (EGCg), a major component of green tea, has been repeatedly reported to have potential therapeutic benefits in HCM. Studies on animal models show that it leads to a decrease in myofilament Ca^2+^ sensitivity [28,29,30], but further studies need to be conducted in order to evaluate if this can furtherly be translated into clinical benefits.

Similarly, Nebivolol is a betablocker that differentiates from the rest of his class due to its ability to also influence Ca^2+^ sensitivity as shown through a study on rabbit and human myocardium, thus making it an agent with potentially two negative inotropy inducing properties [31]. To investigate if these effects could counteract the characteristic HCM hypercontractility, Stucker et al. tested the effect of Nebivolol on contractile parameters of cardiac strips of mouse and human HCM models. Although in HCM mouse strips Nebivolol induced a myofilament Ca^2+^ desensitization, this effect was not observed on human HCM cardiac strips [32].

N-acetylcysteine is an antioxidant that also proved to have a Ca^2+^ desensitizing effect, showed promising results in pre-clinical studies, with a reduction in myocardial oxidative stress and fibrosis as well as a reversal of diastolic dysfunction in mouse models of HCM [33,34,35]. Unfortunately, the results did not translate into clinical trials, as it failed to prove a significant effect on hypertrophy or fibrosis in the HALT-HCM trial [36]. After the attempts at repurposing pre-existing drugs have failed, attention has been redirected towards the development of therapies that would target the pathogenic mechanisms in HCM. The class that has shown results is that of myosin inhibitors, which will be further discussed.

## 3. Myosin Inhibitors—Biomolecular Bases

**Figure 2:**
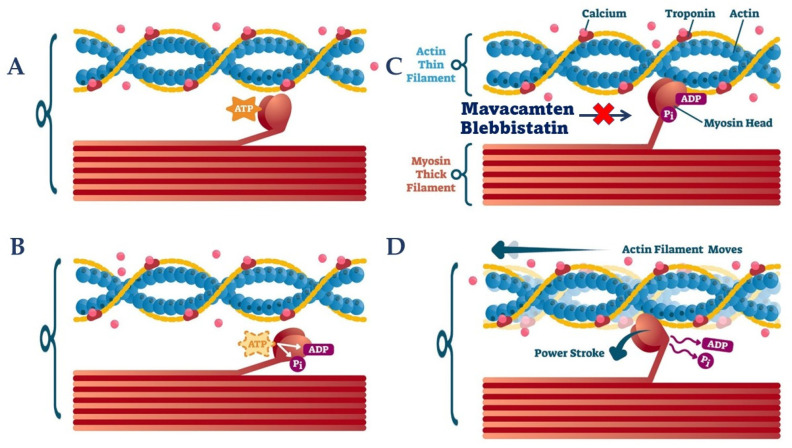
Myosin inhibitors—mechanisms of action. The chemomechanical cycle of myosin is demonstrated in panels (**A**–**D**). (**Panel A**): Binding of ATP to myosin head domain produces a decrease in actin affinity thus leading to actin dissociation (relaxed state). (**Panel B**): ATP hydrolysis leads to formation of ADP and Pi. (**Panel C**): Myosin-ADP-Pi complex binds to actin filaments. (**Panel D**): Conformational changes prompted by Pi release determine the power stroke, with ADP being released at the end of this phase and with a start of a new cycle. **Mavacamten**: (1) inhibits the release of Pi, (2) decreases the number of myosin head that bind to actin. **Blebbistatin**: inhibits Pi release after ATP hydrolysis. ADP = adenosine diphosphate; ATP = adenosine triphosphate; Pi = inorganic phosphate.

The concept of myosin inhibitors is not a new one, with first mentions in literature dating from the 1950s [37]. Myosins are ATP-hydrolysing enzymes that convert chemical energy into mechanical force, using actin as a transport track to produce movement. Their three-part constitution is designed for force generation, with the head/motor domain containing the actin and ATP binding sites, the lever arm amplifying structural changes in the motor domain and the tail being responsible for filament assembly [38,39]. Being that HCM is a hypercontractile disease caused by mutations predominantly of genes that encode MHC-β or cMyBP-C that lead to an altered functionality of the protein [3,4], it is reasonable that attention has been directed towards molecules that would counteract the hypercontractile state (Figure 2).

Blebbistatin is the first widely researched myosin inhibitor after its discovery in 2001 by Cheung et al. [40] It exercises its effects through binding at the myosin head domain while in the relaxed state, while it is in a state of low actin affinity. This leads to the inhibition of phosphate release after ATP hydrolysis, interrupting the force generator chain of events [41,42,43]. The drawbacks in the use of Blebbistatin which lead to abandonment of the idea is its lack of selectivity, as it presents high affinity to skeletal muscle myosin II isoforms and intermediate affinity for cardiac and non-muscle myosin II isoforms, as well as its low potency [44,45]. While with the development of analogues of Blebbistatin problems like potency, water solubility and photosensitivity have been reduced, the lack of sensitivity amongst myosin II isoforms remains unsolved [46].

The first myosin inhibitor to surpass the in-vitro phase studies was Mavacamten (formerly known as MYK-461), a molecule yielded after a chemical screening for compounds that would reduce actin-activated ATPase rate. Initial transient kinetic experiments confirmed that Mavacamten use reduced the rate of phosphate release (the rate-limiting step in the chemo-mechanical force-generating cycle), thus increasing the relaxed state of myosin [47]. Kawas et al. have later hypothesized that Mavacamten acts at multiple stages of the myosin chemo-mechanical cycle, with a secondary mechanism involving the decrease of the number of myosin-S1 heads that interact with the actin filament during transition from the weakly to the strongly-bound state [48].

In subsequent in vivo studies when orally administered to normal and HCM mice, Mavacamten demonstrated a dose-dependent cardiac contractility reduction, without impairment of skeletal muscle function [47].

In humans it has been proven that MYH7 and MYBPC3 genotype positive patients present altered myocardial relaxation and a hyperdynamic contractile status even before LV hypertrophy development [49,50,51]. Green et al. showed that when administered in young pre-hypertrophic HCM mice Mavacamten was associated with a lack of progression of LV hypertrophy compared to placebo-treated HCM mice. Moreover, they observed that in older HCM mice it promoted partial regression of hypertrophy [47].

Similarly, in feline HCM models, Mavacamten treatment reduced cardiac contractility, as evaluated through a decrease in fractional shortening (from 52 ± 3% to 38 ± 7%, *p* = 0.01) without affecting heart rate, with a concomitant relief in LVOT obstruction (*p* = 0.0007) [52].

These encouraging results opened the pathway to the next stage, that of clinical trials.

### 3.1. Applicability in Obstructive HCM

The first trial in humans designed for proof of concept of utilization of Macavamten in individuals with obstructive HCM (oHCM) was phase 2 study PIONEER-HCM, with secondary objectives consisting in characterising pharmacodynamics, pharmacokinetics, as well as safety and tolerability. It included two cohorts that were monitored-up throughout 14 weeks of treatment, followed by 4 weeks in post treatment. Cohort A, designed for proof of concept, included patients who were assigned to receive 10 or 15 mg of Mavacamten daily, according to their body weight (less or over 60 kg, respectively). All betablocker, calcium channel blocker or disopyramide treatment was stopped with at least 14 days prior to the first dose of Macavamten. Cohort B included patients with standard betablocker treatment who received smaller doses of treatment [53].

The results were positive, as after 12 weeks of treatment, a substantial reduction of the mean post-exercise LVOT gradient was observed in Cohort A (mean change, −89.5 mmHg (95% CI, − 138.3 to − 40.7 mmHg)), as well as of the resting LVOT gradient (mean change, −48 mmHg (CI, −72 to −23 mmHg)). The same effects were noted in Cohort B, but with smaller reductions in post-exercise (mean change, −25.0 mm Hg (CI, −47.1 to −3.0 mmHg); *p* = 0.020) and resting LVOT gradient (mean change, −49 mm Hg (CI, −83 to −14 mm Hg); *p* = 0.004). Other secondary outcome results included a reduction of the EF by −15% (CI, −23% to −6%) in Cohort A and −6% (CI, −10% to −1%) in Cohort B, (with complete reversibility after treatment discontinuation), improvement of symptoms as quantified through reduction of NYHA class (mean change −0.9 in Cohort A and mean change −1.0 in Cohort B) and increase in peak oxygen consumption (pVO_2_)(mean increase +3.5ml/kg/min in Cohort A and +1.7ml/kg/min in Cohort B).

From a safety profile point of view, Mavacamten was generally well tolerated with most adverse effects being classified as mild (80%) or moderate (19%). The most frequent adverse effects were EF reduction and atrial fibrillation [53].

Following the encouraging results from the PIONEER-HCM trial, a larger randomized, double-blind placebo-controlled phase 3 trial was conducted to further evaluate Mavacamten as treatment of symptomatic obstructive hypertrophic cardiomyopathy (EXPLORER-HCM). Consequently, a total of 251 patients with HCM with a peak LVOT gradient of >50 mmHg and NYHA class II–III symptoms have been randomly assigned for treatment with Mavacamten or placebo over a period of 30 weeks. The participants were subjected every 2 to 4 weeks to evaluations that included blood tests, electrocardiogram and echocardiography. Noteworthy the fact that discontinuation of betablocker or calcium channel blocker treatment was not required, therefore the majority of the participants (92%) were still under treatment with one of these two drug classes during the period of the study [54].

At the end of the follow up period, the primary endpoint (increase in pVO_2_ with >1.5 ml/kg/min and at least one NYHA class reduction or increase in pVO_2_ of >3ml/kg/min without NYHA class worsening) was achieved in 37% of Mavacamten-receiving participants versus 17% placebo receiving patients (+19.4%, 95% CI 8.7–30.1; *p* = 0.0005). Moreover, a complete clinical response defined as downgrading to NYHA I class symptoms or LVOT gradient <30 mmHg was noted in 27% of participants in the Mavacamten group compared to 1% in the placebo group [55].

Difference in peak post-exercise LVOT gradient at week 30 compared to baseline was significantly higher in the Mavacamten group (from 86 mm Hg (95% CI 79.5 to 91.8) to 38 mmHg (32.3 to 44.0)) than the placebo group (from 84 mm Hg (78.4 to 91.0) to 73 mm Hg (67.2 to 79.6)), showing a greater mean reduction by 35.6 mm Hg with Mavacamten (95% CI −43.2 to −28.1; *p* < 0.0001).

A drop of the peak LVOT gradient lower than the 50 mmHg limit required for SRT has been noted in 74% of patient taking Mavacamten compared to 21% in the placebo group, as well as difference in pVO_2_ (+1.4ml/kg/min with Mavacamten group versus -0.1ml/kg/min with placebo) [55]. Given that even after SRT benefits regarding improvements in pVO_2_ are limited [56], this objective remains a challenge in the management of HCM and the question arises however as to if Mavacamten could represent an alternative to SRT.

From a clinical benefit point of view, 65% of the patients in the Mavacamten group experienced a downgrading of at least 1 NYHA class compared to 31% in the placebo group, with an significantly higher improvement of symptoms quantified by the Kansas City Cardiomyopathy Questionnaire-Clinical Summary Score (KCCQ-CSS) and Hypertrophic Cardiomyopathy Symptom Questionnaire Shortness-of-Breath (HCMSQ-SoB) subscore (KCCQ-CCS +9.1, 95% CI 5.5 to 12.7; HCMSQ-SoB −1.8, −2.4 to −1.2; *p* < 0.0001 for both). Other associated effects involved the significant drop in serum NT-proBNP and hs-cTNI levels, important prognostic factors.

The safety profiles and adverse effects rates were similar between the two groups. A reduction of the EF to <50% was noted in seven patients in the Mavacamten group, with complete return to baseline EF after discontinuation. There was no significant impact on blood pressure and heart rate, effects that frequently limit betablocker or calcium channel inhibitor use [55].

A subsequent substudy targeted the evaluation of structural and functional modifications on cardiac magnetic resonance with Mavacamten. This analysis that included 35 obstructive HCM patients reported a significant reduction in LV mass index in Mavacamten group compared with placebo (mean between-group difference, −15.8 g/m^2^ (95% CI, −22.6 to −9.0); *p* < 0.0001), as well as of the maximum LV wall thickness (mean between-group difference −2.4mm (95% CI −3.9, −0.9); *p* = 0.0079) and of the left atrial volume index index (mean between-group difference, −10.3 mL/m2 (95% CI, −16.0 to −4.6); *p* = 0.0004) [57]. Besides quantifying the anatomic substrate for decreases in LVOT obstruction and symptoms improvements, these parameters are also known to be associated with poor prognosis, making the results even more noteworthy [58,59]. At the same time, myocardial contractile function remained within normal limits and no changes have been observed regarding myocardial fibrosis. The reduction in LV hypertrophy and left atrial volume index have been also shown to be corelated with a decrease in myocardial stress and injury biomarkers [57].

### 3.2. Applicability in Non-Obstructive HCM

Whereas treatment can be escalated in the case of patients with obstructive HCM, with septal reduction therapy with or without mitral valve surgery remaining as a last resort, there are approximately a third of HCM cases that do not present LVOT obstruction [1]. The question remains as to how we treat this category of patients, as treatment options are limited, targeting mostly the control of the heart rate with the help of betablockers and non-dihydropyridine calcium channel blockers. Cardiac transplantation is the only next step available for patients with drug-refractory symptoms [7,8].

Besides the primary mechanism of attenuation of the LVOT obstruction by decreasing contractility, studies have shown that there are complementary pathways through which Mavacamten exerts its benefits. There is now mechanistic evidence that Mavacamten can stabilize the super-relaxed state of β-cardiac myosin, with in vivo studies showing that this type of myosin modulation leads to diminished impaired ventricular filling and improved myocardial energetics [48,60,61].

Due to these complementary pathways activated by Mavacamten it has been speculated that it could be beneficial for non-obstructive HCM, which led to the double-blind placebo-controlled phase 2 study MAVERICK-HCM whose objectives were the evaluation of the safety, tolerability and dose-dependent effects of Mavacamten in symptomatic patients with nonobstructive HCM. A total of 59 patients have been enrolled and randomized 1:1:1 to receive either Mavacamten for a serum concentration of 200ng/mL, for a serum concentration of 500ng/mL or placebo, with a follow up period of 16 weeks during treatment administration followed by an 8-week washout period [62].

The study met its primary objective regarding the safety and tolerability, as no significant difference has been observed between the rate of serious adverse events in the Mavacamten groups (10%) compared to placebo (21%). Amongst the serious adverse events, atrial fibrillation was the most prevalent, both with similar rates between the two groups at around 5%. Although the overall change in LVEF was minor (−4.1 ± 8.0% in the pooled Mavacamten group and −2.3 ± 4.9% in the placebo group), drug administration was discontinued in five participants of the Mavacamten groups due to a decrease in LVEF to <45%, with recovery of the baseline LVEF between 4–12 weeks after discontinuation.

Secondary outcomes analyses revealed significant decreases in biomarkers associated with increased wall stress and myocardial injury, biomarkers that have previously proven to be related to ongoing myocardial fibrosis as well as independent predictors of morbidity and mortality in HCM patients [58,63,64] (NT-proBNP decrease of 53% versus a decrease of 1% with placebo, and a cTnI decrease of 34% versus a 4% increase in the placebo group) [62].

Even though the study was not designed and powered for clinical benefit assessment, exploratory analyses included echocardiography parameters of diastolic function (E/e’, e’ velocity) and a composite functional endpoint (defined as an improvement of at least 1.5 ml/kg/min in pVO_2_ and a reduction of ≥1 NYHA functional class or an improvement of ≥3.0 ml/kg/min in pVO_2_ with no worsening of NYHA functional class). No significant difference was observed in either of these endpoints.

With these results in mind, a phase 3 follow-on study of Mavacamten in nonobstructive HCM is yet necessary in order to assess a potential clinical benefit (all studies involving myosin inhibitors are synthetized in Table 1).

## 4. Discussions and Future Directions

This past decade, we have witnessed tremendous progress regarding the understanding of molecular mechanisms that underlie HCM pathogenesis. This has led to a shift in perspective towards the pharmacological management, with multiple attempts at moving away from drug therapies that manage symptoms and complications towards options that could alter natural history of the cardiac remodelling involved in HCM. After many trial and error attempts, we finally have a potential hope through the class of myosin inhibitors.

With Mavacamten as a first-in-class agent, we have seen positive effects that suggest a reversal of structural modifications with improvements in functional capacity, but many questions remain. Probably the most intriguing aspect that remains to be clarified is where exactly Mavacamten would fit in our day-to-day practice. With this question in mind, the authors of the ongoing study VALOR-HCM (NCT04349072) attempt to provide an answer, as it is as study whose main objective is to assess if treatment with Mavacamten could reduce the number of SRT procedures as this would majorly impact the current treatment algorithm of symptomatic obstructive HCM. This study is expected to be completed at the end of 2024.

At the same time, taking into account that mavamten is a fairly recent addition to our therapeutic arsenal, its long-term adverse effects and safety profile are being investigated throughout the MAVA-LTE extension study of Mavacamten-receiving patients that completed MAVERICK-HCM or EXPLORER-HCM (NCT03723655) as well as the PIONEER-OLE extension trial of PIONEER-HCM (NCT03496168). Initial one-year results have been announced for PIONEER-OLE, stating persistent significant improvements in symptoms, LVOT gradient (at rest: mean 67 ± 42.8 at baseline to 15.3 ± 11.4 at week 48; *p* = 0.0313), NT-proBNP (mean 1836 ± 2886 mmHg at baseline to 206 ± 129 at week 48; *p* = 0.0625) as well as reductions in interventricular septum thickness (mean from 16.7 ± 2.8 at baseline to 14.4 ± 2.8 at week 48, *p* = 0.0313) and indexed left atrial volume (mean 40.9 ± 16.4 to 34.5 ± 7.1, *p* = 0.0313) [65].

Another aspect is related to the most beneficial timing of administration. Given that early use of Mavacamten in animal models has also been proven to halt progression of LV hypertrophy and myocardial fibrosis as well as provide a regression of hypertrophy in older mice [47], the question arises if it could prevent development of phenotypic expression in genotype positive patients. This effect is yet to be investigated in humans.

Other molecules of the same class are currently also under investigation. CK-3773274 (CK-274) is a next-generation cardiac myosin inhibitor that was proven in preclinical models to bind directly to cardiac myosin at a distinct and selective allosteric binding site in a dose–dependent manner. The multicentric, randomized, double-blind, placebo-controlled, dose-finding REDWOOD-HCM trial (Randomized Evaluation of Dosing With CK-274 in Obstructive Outflow Disease in HCM; NCT04219826) announced earlier this year preliminary reports after completion of Cohort 1 phase with the interim analysis showing that patients experienced significant reduction in resting and post-Valsalva LVOT gradients with only modest reductions in LVEF [66].

At the same time, with the favourable results shown by the combination Sacubitril/Valsartan in HF with reduced EF, there are currently studies meant to assess if this drug combination could improve exercise capacity while being safe and well tolerated in patient with non-obstructive HCM (NCT04164732). SILICOFCM (NCT03832660) is another ongoing study meant to evaluate the effect of lifestyle (physical activity and dietary supplementation with inorganic nitrate) and pharmacological (Sacubitril/Valsartan) interventions in patients with HCM (with focus on functional capacity, clinical phenotypic characteristics and quality of life).

## 5. Conclusions

In conclusion, HCM is a complex disease whose pharmacological management remains a challenge for cardiologists, options being currently limited to nonspecific medication like betablockers or calcium channel inhibitors, with frequent suboptimal results. Drugs like Spironolactone and ARBs with aim of antifibrotic effects and reverse-remodeling, as well as myocardial metabolism modulators and late sodium current inhibitors failed to prove a benefit in extensive clinical trials. Some other molecules like Ca^2+^ desensitizers have yet to be studied in clinical trials. However, with advances in comprehension of molecular pathogenesis, opportunities for a targeted treatment arise. That is the case of Mavacamten, first-in-class myosin inhibitor to prove significant results in oHCM with net reduction in LVOT gradient, cardiac biomarkers, and functional parameters, as well as structural improvements at echocardiography and MRI. We expect many studies in the years to come that will try to validate the value of myosin inhibitors and to establish where they will fit in the HCM treatment algorithm.

## Figures and Tables

**Table 1:** Summary of clinical trials involving myosin inhibitors.

Study	Type of Study	Population/ Intervention	Primary Endpoint	Key Findings
**PIONEER-HCM** [53]	Phase II open-label study	21 symptomatic oHCM: Cohort A: 10–20mg Mavacamten Cohort B: 2–5mg Mavacamten	Post-exercise LVOT gradient	Significant decrease of LVOT gradient (−89.5 mmHg in Cohort A and −25.0 mmHg in Cohort B) Secondary outcomes: - increase in pVO2 - well tolerated
**EXPLORER-HCM** [55]	Phase III RCT	251 symptomatic oHCM patients: Mavacamten vs. Placebo	- Increase in pVO_2_ with >1.5ml/kg/min and at least one NYHA class reduction Or - Increase in pVO_2_ of >3ml/kg/min without NYHA class worsening	Primary outcome: 37% vs. 17% of patients (Mavacamten, respectively placebo) Secondary outcomes: - greater reductions in post-exercise LVOT gradient - greater increase in pVO2 - improved symptom scores
**MAVERICK-HCM** [62]	Phase II RCT	59 symptomatic non-oHCM Mavacamten vs. Placebo	Frequency and severity of adverse events	No significant difference in the rate of serious adverse events Secondary outcomes: - important reduction of NT-proBNP and cTnI
**PIONEER-OLE** [65]	Phase II open label extension study	20 (estimated enrollment) Mavacamten as in PIONEER- HCM	Frequency and severity of adverse events up to 260 weeks	Intermediate results at 1 year: - Persistent decrease in LVOT gradient, NT-proBNP, IVS and LAVI - well tolerated
**MAVA-LTE** **(NCT03723655)**	Phase II and III open label extension study	310 (estimated enrollment) Mavacamten as in EXPLORER-HCM and MAVERICK-HCM	Frequency and severity of adverse events up to 252 weeks	Ongoing study
**VALOR-HCM** **(NCT04349072)**	Phase III RCT	100 (estimated enrollment): Mavacamten vs. Placebo	No of subjects who remain guideline eligible for SRT at Week 16	Ongoing study
**REDWOOD-HCM** **(NCT04219826)**	Phase II RCT	60 (estimated enrollment) CK-3773274	Incidence of reported adverse events	Ongoing study

IVS = interventricular septum thickness; LAVI = indexed left atrial volume; LVOT = left ventricle outflow tract; oHCM = obstructive hypertrophic cardiomyopathy; pVO2 = peak oxygen consumption; RCT = randomized controlled trial; SRT = septal reduction therapy.

## References

[1] Marian A.J., Braunwald E. (2017). Hypertrophic Cardiomyopathy. Circ. Res..

[2] Maron B.J. (2018). Clinical Course and Management of Hypertrophic Cardiomyopathy. N. Engl. J. Med..

[3] Richard P., Charron P., Carrier L., Ledeuil C., Cheav T., Pichereau C., Benaiche A., Isnard R., Dubourg O., Burban M. (2003). Hypertrophic Cardiomyopathy. Circulation.

[4] Bos J.M., Will M.L., Gersh B.J., Kruisselbrink T.M., Ommen S.R., Ackerman M.J. (2014). Characterization of a Phenotype-Based Genetic Test Prediction Score for Unrelated Patients With Hypertrophic Cardiomyopathy. Mayo Clin. Proc..

[5] Geske J.B., Ommen S.R., Gersh B.J. (2018). Hypertrophic Cardiomyopathy. JACC Heart Fail..

[6] Ho C.Y., Day S.M., Ashley E.A., Michels M., Pereira A.C., Jacoby D., Cirino A.L., Fox J.C., Lakdawala N.K., Ware J.S. (2018). Genotype and Lifetime Burden of Disease in Hypertrophic Cardiomyopathy. Circulation.

[7] Elliott P.M., Anastasakis A., Borger M.A., Borggrefe M., Cecchi F., Charron P., Hagege A.A., Lafont A., Limongelli G., Mahrholdt H. (2014). 2014 ESC Guidelines on diagnosis and management of hypertrophic cardiomyopathy: The Task Force for the diagnosis and management of hypertrophic cardiomyopathy of the European Society of Cardiology (ESC). Eur. Heart J..

[8] Ommen S.R., Mital S., Burke M.A., Day S.M., Deswal A., Elliott P., Evanovich L.L., Hung J., Joglar J.A., Kantor P. (2020). 2020 AHA/ACC Guideline for the Diagnosis and Treatment of Patients With Hypertrophic Cardiomyopathy. Circulation.

[9] Wells S., Rowin E.J., Boll G., Rastegar H., Wang W., Maron M.S., Maron B.J. (2018). Clinical Profile of Nonresponders to Surgical Myectomy with Obstructive Hypertrophic Cardiomyopathy. Am. J. Med..

[10] Spoladore R., Maron M.S., D’Amato R., Camici P.G., Olivotto I. (2012). Pharmacological treatment options for hypertrophic cardiomyopathy: High time for evidence. Eur. Heart J..

[11] Maron M.S., Chan R.H., Kapur N.K., Jaffe I.Z., McGraw A.P., Kerur R., Maron B.J., Udelson J.E. (2018). Effect of Spironolactone on Myocardial Fibrosis and Other Clinical Variables in Patients with Hypertrophic Cardiomyopathy. Am. J. Med..

[12] Marian A. (2000). Pathogenesis of diverse clinical and pathological phenotypes in hypertrophic cardiomyopathy. Lancet.

[13] Lim D.-S., Lutucuta S., Bachireddy P., Youker K., Evans A., Entman M., Roberts R., Marian A.J. (2001). Angiotensin II Blockade Reverses Myocardial Fibrosis in a Transgenic Mouse Model of Human Hypertrophic Cardiomyopathy. Circulation.

[14] Shimada Y., Passeri J.J., Baggish A.L., O’Callaghan C., Lowry P.A., Yannekis G., Abbara S., Ghoshhajra B., Rothman R.D., Ho C.Y. (2013). Effects of Losartan on Left Ventricular Hypertrophy and Fibrosis in Patients With Nonobstructive Hypertrophic Cardiomyopathy. JACC Heart Fail..

[15] Axelsson A., Iversen K., Vejlstrup N., Ho C., Norsk J., Langhoff L., Ahtarovski K., Corell P., Havndrup O., Jensen M.K. (2015). Efficacy and safety of the angiotensin II receptor blocker losartan for hypertrophic cardiomyopathy: The INHERIT randomised, double-blind, placebo-controlled trial. Lancet Diabetes Endocrinol..

[16] Axelsson A., Iversen K., Vejlstrup N., Ho C.Y., Havndrup O., Kofoed K., Norsk J., Jensen M.K., Bundgaard H. (2016). Functional effects of losartan in hypertrophic cardiomyopathy—A randomised clinical trial. Heart.

[17] Raja A.A., Shi L., Day S.M., Russell M., Zahka K., Lever H., Colan S.D., Margossian R., Hall E.K., Becker J. (2019). Baseline Characteristics of the VANISH Cohort. Circ. Heart Fail..

[18] Coppini R., Ferrantini C., Yao L., Fan P., Del Lungo M., Stillitano F., Sartiani L., Tosi B., Suffredini S., Tesi C. (2013). Late Sodium Current Inhibition Reverses Electromechanical Dysfunction in Human Hypertrophic Cardiomyopathy. Circulation.

[19] Coppini R., Mazzoni L., Ferrantini C., Gentile F., Pioner J.M., Laurino A., Santini L., Bargelli V., Rotellini M., Bartolucci G. (2017). Ranolazine Prevents Phenotype Development in a Mouse Model of Hypertrophic Cardiomyopathy. Circ. Heart Fail..

[20] Olivotto I., Camici P.G., Merlini P.A., Rapezzi C., Patten M., Climent V., Sinagra G., Tomberli B., Marin F., Ehlermann P. (2018). Efficacy of Ranolazine in Patients With Symptomatic Hypertrophic Cardiomyopathy. Circ. Heart Fail..

[21] Olivotto I., Hellawell J.L., Farzaneh-Far R., Blair C., Coppini R., Myers J., Belardinelli L., Maron M.S. (2016). Novel Approach Targeting the Complex Pathophysiology of Hypertrophic Cardiomyopathy. Circ. Heart Fail..

[22] Crilley J.G., A Boehm E., Blair E., Rajagopalan B., Blamire A.M., Styles P., McKenna W.J., Östman-Smith I., Clarke K., Watkins H. (2003). Hypertrophic cardiomyopathy due to sarcomeric gene mutations is characterized by impaired energy metabolism irrespective of the degree of hypertrophy. J. Am. Coll. Cardiol..

[23] Gehmlich K., Dodd M., Allwood J.W., Kelly M., Bellahcene M., Lad H.V., Stockenhuber A., Hooper C., Ashrafian H., Redwood C.S. (2014). Changes in the cardiac metabolome caused by perhexiline treatment in a mouse model of hypertrophic cardiomyopathy. Mol. BioSyst..

[24] Abozguia K., Elliott P., McKenna W.J., Phan T.T., Nallur-Shivu G., Ahmed I., Maher A.R., Kaur K., Taylor J., Henning A. (2010). Metabolic Modulator Perhexiline Corrects Energy Deficiency and Improves Exercise Capacity in Symptomatic Hypertrophic Cardiomyopathy. Circulation.

[25] Coats C.J., Pavlou M., Watkinson O.T., Protonotarios A., Moss L., Hyland R., Rantell K., Pantazis A.A., Tome M., McKenna W.J. (2019). Effect of Trimetazidine Dihydrochloride Therapy on Exercise Capacity in Patients With Nonobstructive Hypertrophic Cardiomyopathy. JAMA Cardiol..

[26] Baudenbacher F., Schober T., Pinto J.R., Sidorov V.Y., Hilliard F., Solaro R.J., Potter J.D., Knollmann B.C. (2008). Myofilament Ca2+ sensitization causes susceptibility to cardiac arrhythmia in mice. J. Clin. Investig..

[27] Alves M.L., Dias F.A., Gaffin R.D., Simon J.N., Montminy E.M., Biesiadecki B.J., Hinken A.C., Warren C.M., Utter M.S., Davis R.T. (2014). Desensitization of myofilaments to Ca^2+^ as a therapeutic target for hypertrophic cardiomyopathy with mutations in thin filament proteins. Circ. Cardiovasc. Genet..

[28] Tadano N., Du C.-K., Yumoto F., Morimoto S., Ohta M., Xie M.-F., Nagata K., Zhan N.-Y., Lu Q.-W., Miwa Y. (2010). Biological actions of green tea catechins on cardiac troponin C. Br. J. Pharmacol..

[29] Warren C.M., Karam C.N., Wolska B.M., Kobayashi T., De Tombe P.P., Arteaga G.M., Bos J.M., Ackerman M.J., Solaro R.J. (2015). Green Tea Catechin Normalizes the Enhanced Ca2+ Sensitivity of Myofilaments Regulated by a Hypertrophic Cardiomyopathy–Associated Mutation in Human Cardiac Troponin I (K206I). Circ. Cardiovasc. Genet..

[30] Friedrich F.W., Flenner F., Nasib M., Eschenhagen T., Carrier L. (2016). Epigallocatechin-3-Gallate Accelerates Relaxation and Ca2+ Transient Decay and Desensitizes Myofilaments in Healthy and Mybpc3-Targeted Knock-in Cardiomyopathic Mice. Front. Physiol..

[31] Zeitz O., Rahman A., Hasenfuss G., Janssen P.M. (2000). Impact of β-Adrenoceptor Antagonists on Myofilament Calcium Sensitivity of Rabbit and Human Myocardium. J. Cardiovasc. Pharmacol..

[32] Stücker S., Kresin N., Carrier L., Friedrich F.W. (2017). Nebivolol Desensitizes Myofilaments of a Hypertrophic Cardiomyopathy Mouse Model. Front. Physiol..

[33] Marian A.J., Senthil V., Chen S.N., Lombardi R. (2006). Antifibrotic Effects of Antioxidant N-Acetylcysteine in a Mouse Model of Human Hypertrophic Cardiomyopathy Mutation. J. Am. Coll. Cardiol..

[34] Lombardi R., Rodriguez G., Chen S.N., Ripplinger C.M., Li W., Chen J., Willerson J.T., Betocchi S., Wickline S.A., Efimov I. (2009). Resolution of Established Cardiac Hypertrophy and Fibrosis and Prevention of Systolic Dysfunction in a Transgenic Rabbit Model of Human Cardiomyopathy Through Thiol-Sensitive Mechanisms. Circulation.

[35] Wilder T., Ryba D., Wieczorek D.F., Wolska B.M., Solaro R.J. (2015). N-acetylcysteine reverses diastolic dysfunction and hypertrophy in familial hypertrophic cardiomyopathy. Am. J. Physiol. Circ. Physiol..

[36] Marian A.J., Tan Y., Li L., Chang J.T., Syrris P., Hessabi M., Rahbar M.H., Willerson J.T., Cheong B.Y., Liu C.-Y. (2018). Hypertrophy Regression With N-Acetylcysteine in Hypertrophic Cardiomyopathy (HALT-HCM). Circ. Res..

[37] Wilson I.B., Ginsburg S. (1955). A powerful reactivator of alkylphosphate-inhibited acetylcholinesterase. Biochim. Biophys. Acta Bioenerg..

[38] Sellers J.R. (2000). Myosins: A diverse superfamily. Biochim. Biophys. Acta Bioenerg..

[39] Sweeney H.L., Houdusse A. (2010). Structural and Functional Insights into the Myosin Motor Mechanism. Annu. Rev. Biophys..

[40] Cheung A., Dantzig J.A., Hollingworth S., Baylor S.M., Goldman Y., Mitchison T.J., Straight A.F. (2001). A small-molecule inhibitor of skeletal muscle myosin II. Nat. Cell Biol..

[41] Ramamurthy B., Yengo C.M., Straight A.F., Mitchison T.J., Sweeney H.L. (2004). Kinetic Mechanism of Blebbistatin Inhibition of Nonmuscle Myosin IIB. Biochem..

[42] Kovács M., Tóth J., Hetényi C., Malnasi-Csizmadia A., Sellers J.R. (2004). Mechanism of Blebbistatin Inhibition of Myosin II. J. Biol. Chem..

[43] Allingham J.S., Smith R., Rayment I. (2005). The structural basis of blebbistatin inhibition and specificity for myosin II. Nat. Struct. Mol. Biol..

[44] Straight A.F., Cheung A., Limouze J., Chen I., Westwood N., Sellers J.R., Mitchison T.J. (2003). Dissecting Temporal and Spatial Control of Cytokinesis with a Myosin II Inhibitor. Science.

[45] Limouze J., Straight A.F., Mitchison T., Sellers J.R. (2004). Specificity of blebbistatin, an inhibitor of myosin II. J. Muscle Res. Cell Motil..

[46] Roman B.I., Verhasselt S., Stevens C.V. (2018). Medicinal Chemistry and Use of Myosin II Inhibitor (S)-Blebbistatin and Its Derivatives. J. Med. Chem..

[47] Green E., Wakimoto H., Anderson R.L., Evanchik M.J., Gorham J.M., Harrison B.C., Henze M., Kawas R., Oslob J.D., Rodriguez H.M. (2016). A small-molecule inhibitor of sarcomere contractility suppresses hypertrophic cardiomyopathy in mice. Science.

[48] Kawas R.F., Anderson R.L., Ingle S.R.B., Song Y., Sran A.S., Rodriguez H.M. (2017). A small-molecule modulator of cardiac myosin acts on multiple stages of the myosin chemomechanical cycle. J. Biol. Chem..

[49] Ho C.Y., Sweitzer N.K., McDonough B., Maron B.J., Casey S.A., Seidman J., Seidman C.E., Solomon S.D. (2002). Assessment of Diastolic Function With Doppler Tissue Imaging to Predict Genotype in Preclinical Hypertrophic Cardiomyopathy. Circulation.

[50] Forsey J., Benson L., Rozenblyum E., Friedberg M.K., Mertens L. (2014). Early Changes in Apical Rotation in Genotype Positive Children with Hypertrophic Cardiomyopathy Mutations without Hypertrophic Changes on Two-Dimensional Imaging. J. Am. Soc. Echocardiogr..

[51] Rüssel I.K., Brouwer W.P., Germans T., Knaapen P., Marcus T.J., Van Der Velden J., Götte M.J., Van Rossum A.C. (2011). Increased left ventricular torsion in hypertrophic cardiomyopathy mutation carriers with normal wall thickness. J. Cardiovasc. Magn. Reson..

[52] Stern J.A., Markova S., Ueda Y., Kim J.B., Pascoe P., Evanchik M.J., Green E.M., Harris S.P. (2016). A Small Molecule Inhibitor of Sarcomere Contractility Acutely Relieves Left Ventricular Outflow Tract Obstruction in Feline Hypertrophic Cardiomyopathy. PLoS ONE.

[53] Heitner S.B., Jacoby D., Lester S.J., Owens A., Wang A., Zhang D., Lambing J., Lee J., Semigran M., Sehnert A.J. (2019). Mavacamten Treatment for Obstructive Hypertrophic Cardiomyopathy. Ann. Intern. Med..

[54] Ho C.Y., Olivotto I., Jacoby D., Lester S.J., Roe M., Wang A., Waldman C.B., Zhang D., Sehnert A.J., Heitner S.B. (2020). Study Design and Rationale of EXPLORER-HCM. Circ. Heart Fail..

[55] Olivotto I., Oreziak A., Barriales-Villa R., Abraham T.P., Masri A., Garcia-Pavia P., Saberi S., Lakdawala N.K., Wheeler M.T., Owens A. (2020). Mavacamten for treatment of symptomatic obstructive hypertrophic cardiomyopathy (EXPLORER-HCM): A randomised, double-blind, placebo-controlled, phase 3 trial. Lancet.

[56] Smith J.R., Layrisse V., Medina-Inojosa J.R., Berg J.D., Ommen S.R., Olson T.P. (2020). Predictors of exercise capacity following septal myectomy in patients with hypertrophic cardiomyopathy. Eur. J. Prev. Cardiol..

[57] Saberi S., Cardim N., Yamani M.H., Schulz-Menger J., Li W., Florea V., Sehnert A.J., Kwong R.Y., Jerosch-Herold M., Masri A. (2021). Mavacamten Favorably Impacts Cardiac Structure in Obstructive Hypertrophic Cardiomyopathy. Circulation.

[58] Geske J.B., McKie P.M., Ommen S.R., Sorajja P. (2013). B-Type Natriuretic Peptide and Survival in Hypertrophic Cardiomyopathy. J. Am. Coll. Cardiol..

[59] Sascău R., Zota I.M., Stătescu C., Boișteanu D., Roca M., Maștaleru A., Constantin M.M.L., Vasilcu T.F., Gavril R.S., Mitu F. (2018). Review of Echocardiographic Findings in Patients with Obstructive Sleep Apnea. Can. Respir. J..

[60] Del Rio C.L., Ueyama Y., Dale B., Dalton S., Laurence L., Philip J., Olivier B., Lambing J., Evanchik M.J., Green E.M. (2017). Abstract 20593: In vivo Cardiac Effects of Mavacamten (MYK-461): Evidence for Negative Inotropy and Improved Compliance. Circulation.

[61] Anderson R.L., Trivedi D.V., Sarkar S.S., Henze M., Ma W., Gong H., Rogers C.S., Gorham J.M., Wong F.L., Morck M.M. (2018). Deciphering the super relaxed state of human β-cardiac myosin and the mode of action of mavacamten from myosin molecules to muscle fibers. Proc. Natl. Acad. Sci. USA.

[62] Ho C.Y., Mealiffe M.E., Bach R.G., Bhattacharya M., Choudhury L., Edelberg J.M., Hegde S.M., Jacoby D., Lakdawala N.K., Lester S.J. (2020). Evaluation of Mavacamten in Symptomatic Patients With Nonobstructive Hypertrophic Cardiomyopathy. J. Am. Coll. Cardiol..

[63] Kawasaki T., Sakai C., Harimoto K., Yamano M., Miki S., Kamitani T. (2013). Usefulness of High-Sensitivity Cardiac Troponin T and Brain Natriuretic Peptide as Biomarkers of Myocardial Fibrosis in Patients with Hypertrophic Cardiomyopathy. Am. J. Cardiol..

[64] Bostan M.-M., Stătescu C., Anghel L., Șerban I.-L., Cojocaru E., Sascău R. (2020). Post-Myocardial Infarction Ventricular Remodeling Biomarkers—The Key Link between Pathophysiology and Clinic. Biomolecules.

[65] Heitner S.B., Lester S., Wang A., Hegde S.M., Fang L., Balaratnam G., Sehnert A.J., Jacoby D. (2019). Abstract 13962: Precision Pharmacological Treatment for Obstructive Hypertrophic Cardiomyopathy with Mavacamten: One-Year Results From PIONEER-OLE. Circulation.

[66] Cytokinetics Announces Progression of REDWOOD-HCM to Cohort 2. https://www.globenewswire.com/news-release/2020/12/09/2142115/0/en/Cytokinetics-Announces-Progression-of-REDWOOD-HCM-to-Cohort-2.html.

